# Differential effects of mood stabilizers on telomere homeostasis in neuroblastoma cells in vitro

**DOI:** 10.1192/j.eurpsy.2026.10734

**Published:** 2026-07-31

**Authors:** J. Thurin, A. Rathes, L.-A. Marivin, F. Bellivier, B. Etain, C. Marie-Claire

**Affiliations:** 1 Université Paris Cité - INSERM, Optimisation Thérapeutique en Neuropharmacologie - OPTEN U1144; 2 GHU APHP.Nord – Université Paris Cité - Département de Psychiatrie et de Médecine Addictologique- Hôpitaux Lariboisiere-Fernand Widal, Paris; 3 Fondation Fondamental, Créteil, France

## Abstract

**Introduction:**

Telomeres, non-coding DNA sequences protecting the ends of chromosomes, are biomarkers of cellular ageing. Individuals with bipolar disorder (BD) have shorter telomere length (TL) compared to the general population, though the underlying molecular mechanism is unknown. TL homeostasis is regulated by 3 multiprotein complexes: the telomerase holoenzyme, the Shelterin and the CST complex. Lithium (Li) and valproic acid (VPA) are two commonly prescribed mood stabilizers for BD. Three studies has examined the potential protective effect of Li on TL or the expression of TERT (Telomerase reverse transcriptase), the catalytic subunit of telomerase in in-vitro models with conflicting results (Fries *et al.*j.jpsychires.2020),(Squassina *et al.* j.euroneuro.2016)(Squassina *et al*.Hum Genomics.2022). No data are available on other proteins within the three complexes, the measure of telomerase activity(TA) as well as on the effects of VPA.

**Objectives:**

This study aims to evaluate the impact of Li and VPA on the telomere homeostasis system in a neuronal in-vitro model.

**Methods:**

SH-SY5Y neuroblastoma cells were cultured with Li or VPA at therapeutic range concentrations. Cells were collected after 7, 14 and 21 days of exposure. Then, measure of gene expression of 16 genes encoding proteins of the 3 complexes was conducted using RT-qPCR, measure of TERT protein levels using Wester-blot, telomerase activity (TA) analysis using a Telomeric Repeat Amplification Protocol assay and TL analysis using qPCR. Group comparisons were performed using the Kruskal–Wallis test with the R software.

**Results:**

No significant changes were identified between all analysed parameters of cells exposed to Li compared to controls at any time of exposure. However, for VPA, 7 out of 16 genes of the three complexess were altered at D7, D14 or D21 following distincts kinetics, including a downregulation of TERT (p=0.001). Additionally, at D21, TERT protein levels and TA were decreased compared to controls (p=0.02 and p=0.004). However, no changes in TL were observed at any time of exposure.

**Conclusions:**

In the neuroblastoma model Li shows no protective effect on the telomere homeostasis system. VPA negatively impacts telomere homeostasis by limiting TA through the downregulation of TERT and a decrease in TERT protein levels at D21. It also alters the expression of multiple other genes within the three complexes. However, without significant changes in TL. The reduction in TA observed only at D21 suggests that a longer duration may be necessary to affect TL. The presence of alternative telomere lengthening mechanisms in SH-SY5Y cells (activated when TERT is supressed) could also explain these findings. Since the telomere homeostasis system is altered in cancer cells such as SH-SY5Y, similar analyses should be conducted in other in-vitro models with extended exposure durations.

**Disclosure of Interest:**

None Declared

